# DNA-guided photoactivatable probe-based chemical proteomics reveals the reader protein of mRNA methylation

**DOI:** 10.1016/j.isci.2021.103046

**Published:** 2021-08-28

**Authors:** Yepei Huang, Xue Bai, Zhenchang Guo, Hanyang Dong, Yun Fu, Hui Zhang, Guijin Zhai, Shanshan Tian, Ye Wang, Kai Zhang

**Affiliations:** 1 The Province and Ministry Co-sponsored Collaborative Innovation Center for Medical Epigenetics, Key Laboratory of Breast Cancer Prevention and Therapy (Ministry of Education), Key Laboratory of Immune Microenvironment and Disease (Ministry of Education), Department of Biochemistry and Molecular Biology, School of Basic Medical Sciences, Tianjin Medical University Cancer Institute and Hospital, Tianjin Medical University General Hospital, Tianjin Medical University, Tianjin 300070, China; 2 College of Chemistry and Molecular Engineering, Peking University, Beijing 100871, China

**Keywords:** Biochemistry methods, Molecular biology, Proteomics

## Abstract

Chemical modification on mRNA can recruit specific binding proteins (readers/partners) to determine post-transcriptional gene regulation. However, the identification of the reader is extremely limited owing to the rather weak and highly dynamic non-covalent interactions between mRNA modification and reader, and therefore the sensitive and robust approaches are desirable. Here, we report a DNA-guided photoactivatable-based chemical proteomic approach for profiling the readers of mRNA methylation. By use of N^6^-methyladenosine (m^6^A), we illustrated that this method can be successfully utilized for labelling and enriching the readers of mRNA modification, as well as for the discovery of new partners. Thus we applied this strategy to a new modification 2′-O-methyladenosine. As a result, DDX1 was identified and verified as a potential binding protein. Our study therefore provides a powerful chemical proteomics tool for identifying the binding factors of mRNA modification and reveals the underlying function of mRNA modification.

## Highlights

• A DNA-templated probe for covalent capture of the weak RNA-protein interactions
• Selective labelling and enrichment of binding factors to mRNA methylation
• DDX1 was identified as a potential binding protein of 2′-O-methyladenosine
• A chemical proteomics tool for identifying the binding partners of RNA modifications

## Graphical abstract

**Figure:**
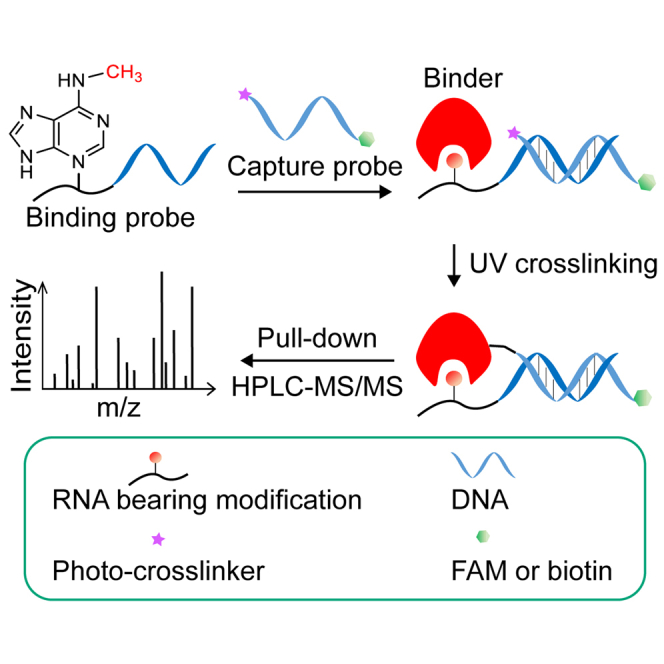

## Introduction

The catalog of posttranscriptional chemical modifications on lowly expressed messenger RNA (mRNAs) has recently been expanded greatly, including N^6^-methyladenosine (m^6^A), 5-methylcytosine (m^5^C), N^1^-methyladenosine (m^1^A), pseudouridine (Ψ), 5-hydroxymethylcytosine (hm^5^C), 2′-O-methylation (Nm) and so on (Boccaletto et al., 2018). To date, the mRNA methylation has attracted much interest because it plays a crucial role in mRNA metabolism (Gilbert et al., 2016), gene regulation (Roundtree et al., 2017), DNA damage response (Xiang et al., 2017), and human diseases (Zhang et al., 2017). It has been believed that the mRNA methylation is dynamically regulated by the protein machinery including ‘writers’ (e.g. METTL3, METTL14, NAT10), ‘erasers’ (e.g. FTO, ALKBH5) and ‘readers’ (e.g. YTH family, ALYREF) to alter cellular process in an epigenetic manner (Lewis et al., 2017). The writers and erasers alter the modification level, whereas the readers specifically recognize and bind to the targeted mRNA modification thus to determine downstream events. These readers have been demonstrated to alter mRNA fate from synthesis to decay (Shi et al., 2019). For examples, for m^6^A readers, YTH domain family 1 (YTHDF1) increases mRNA translation by facilitating ribosome loading, whereas YTHDF2 locates bound mRNA to decay site, a different group of proteins, such as HNRNPC, prefer an “m^6^A-switch” (Liu et al., 2015a), which remodels local RNA structure and consequently modulates RNA-protein interactions around or nearby. Identifying these binding factors will provide us a profound insight into our understanding of the fine-tune regulation by epigenetic modification temporally and spatially, and thus will contribute further to our explanation of the regulation mechanisms and physiological function of mRNA. However, the understanding of mRNA readers is still limited because of low abundance of modification and the rather weak and highly dynamic non-covalent interactions between mRNA modification and readers. It is still challenging to detect such interactions in transcriptomes, therefore the sensitive and robust technologies are urgently desirable.

RNA probe pull-down is a typical approach for the discovery of readers (Edupuganti et al., 2017), but its application is still limited to the characterization of kinetically stable RNA-protein interactions. A definite improvement has been achieved by a photo-crosslinking reaction that converts the unstable non-covalent interactions into the stable covalent bonds (Arguello et al., 2017). However, the attached crosslinking group might cause potential steric hindrance and thus alter the recognized motif in mRNA, which eventually may affect the binding and identification of reader proteins. To resolve the issue, we introduced DNA templated technique that can guide the desired groups in a site-specific manner through the self-assembly of complementary double-stranded DNA, which has been applied in the screening interaction of protein-small molecule (Li et al., 2013), protein-protein (Bai et al., 2016) and protein-DNA (Liu et al., 2015b). As far as structure is concerned, single-stranded mRNA is easier to integrate with DNA than protein or small molecule, and therefore this technique is highly expected to be suitable for the design of mRNA modification probes.

In this study, we have attempted to develop a dual probe-based DNA-templated, photo-affinity crosslinking approach for interrogating the interactome of mRNA methylation, and to describe a systematic unbiased labeling and pull-down method to define the mRNA readers. We illustrated the applicability and effectiveness of the method by identifying known m^6^A binders. Thereby we applied this strategy to Am, and found that ATP-dependent RNA helicase DDX1 (DDX1) binds to Am. Thus our study provides a general strategy to profile the interactome of modified RNA and expands the known repertoire of m^6^A/Am-methylation regulated protein−RNA interactions.

## Results

### Analysis strategy and preparation of DNA-guided photoactivatable RNA probes

**Figure 1:**
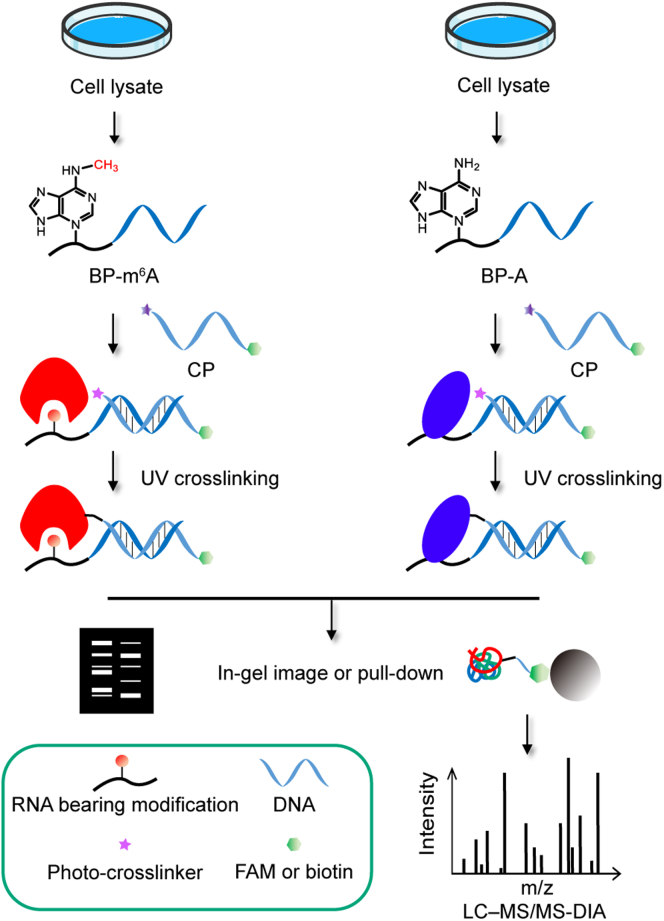
DNA-guided photoactivatable probe-based chemical proteomics for the profiling of the m^6^A interactome BP: binding probe, CP: capture probe.

To profile binding partners of mRNA modification, a dual probe is designed. (i) RNA oligonucleotide bearing mRNA modification acted as a recognizing group is conjugated to a single-stranded DNA to generate a “binding probe” (BP). And (ii) a “capture probe” (CP) is prepared by conjugating a photo-reactive group (diazirine) to another complementary single-stranded DNA that is modified by a biotin or fluorescein (FAM) for detection. A brief description of the strategy is outlined in Figure 1. (1) BP is first incubated with samples to preliminarily recruit proteins that specifically recognize mRNA modification, (2) then the CP is added, the cross-linking groups are brought to a very adjacent position to the binding protein via BP/CP hybridization. (3) A covalent bond is further formed between the capture probe and its target proteins under UV light. (4) The captured proteins can be detected by in-gel imaging with FAM tag or affinity enrichment with biotin tag for mass spectrometry (MS) analysis. To screen the specific binding proteins, the quantitative analysis is carried out by comparing parallel experimental and control groups (the same mRNA sequence without modification). Furthermore, we set up data independent acquisition (DIA) scan mode to obtain more sensitive detection. Finally, the potential readers were verified by western blot assay and MS analysis in parallel reaction monitoring (PRM) scan mode to monitor the binding between candidate proteins and RNA modification (Table S4).

### Analytical performance of the DNA-guided photoactivatable RNA probe

It has been thought that m^6^A methylation as the best-characterized internal mRNA modification regulates gene expression through the direct recognition and downstream effect of m^6^A readers (Zhu et al., 2014). Herein, we designed RNA probes containing the m^6^A consensus sequence GGACU and its reading domain YTH of YTHDF2 (Figure S1A) as a representative to prepare probes and optimize experimental conditions.

**Figure 2:**
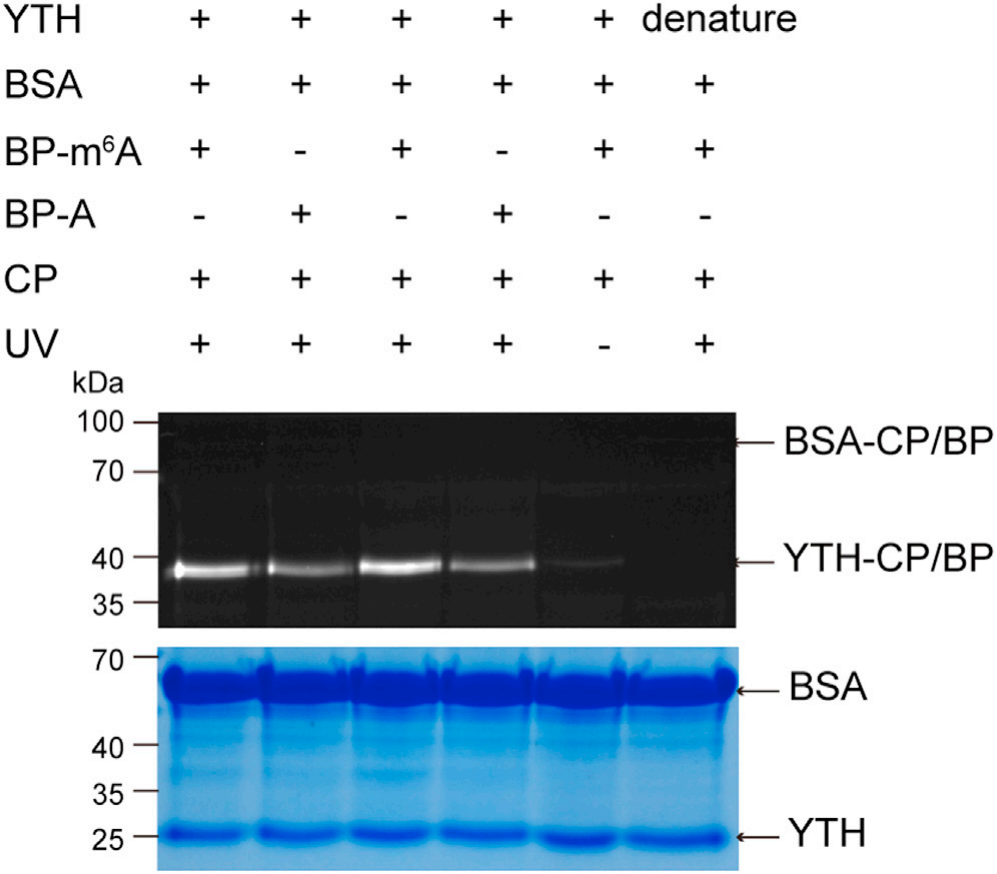
*In vitro* labeling of YTHDF2-YTH by the RNA probe in the presence of BSA Lane 1 and 3: experiment performed in the presence of BP, CP, YTH, and with UV irradiation. Lane 2 and 4: no m^6^A modification (BP-A). Lane 1 and 2: YTH and dual probe were mixed and incubated all at once. Lane 3 and 4: YTH and BP were pre-incubated for 1 hr and then co-incubated with CP for 1 hr. Lane 5: no UV irradiation. Lane 6: YTH was SDS-denatured. YTH = 3 μM, BP/CP-FAM = 12 μM. BSA = 3 μM.

Firstly, we tested whether the strategy could be applied to the detection of RNA-protein interactions through a group of fluorescence labeling experiments. We prepared the m^6^A probe and corresponding unmodified probe as a control, and incubated them with YTH domain in the presence of BSA, respectively. After UV excitation and electrophoretic separation, the labelled proteins were visible via in-gel imaging. As shown in Figure 2, even though plentiful nonspecific BSA was present, YTH domain was successfully labelled by m^6^A RNA probe (lane 1), which suggested that the probe has a good specificity. The negative controls produced no obvious YTH labeling (lane 5: no UV irradiation, lane 6: YTH was denatured), indicating the importance of effective UV-crosslinking for trapping the target and the active protein for recognition of probes. Compared with the control, m^6^A probe has a better performance in trapping the target (lane 1 and 2). In addition, whether the CP was attached to BP or not, the binding affinity is not obviously changed (lane 1 and lane 3). It indicates that crosslinker or tag on CP has little effect on the binding between BP and target proteins, and the introduction of this modified group causes little steric hindrance, thus illustrating the flexibility of the probe. All the above observations convinced us that the strategy can be used for reliable and specific detection of m^6^A binders.

**Figure 3:**
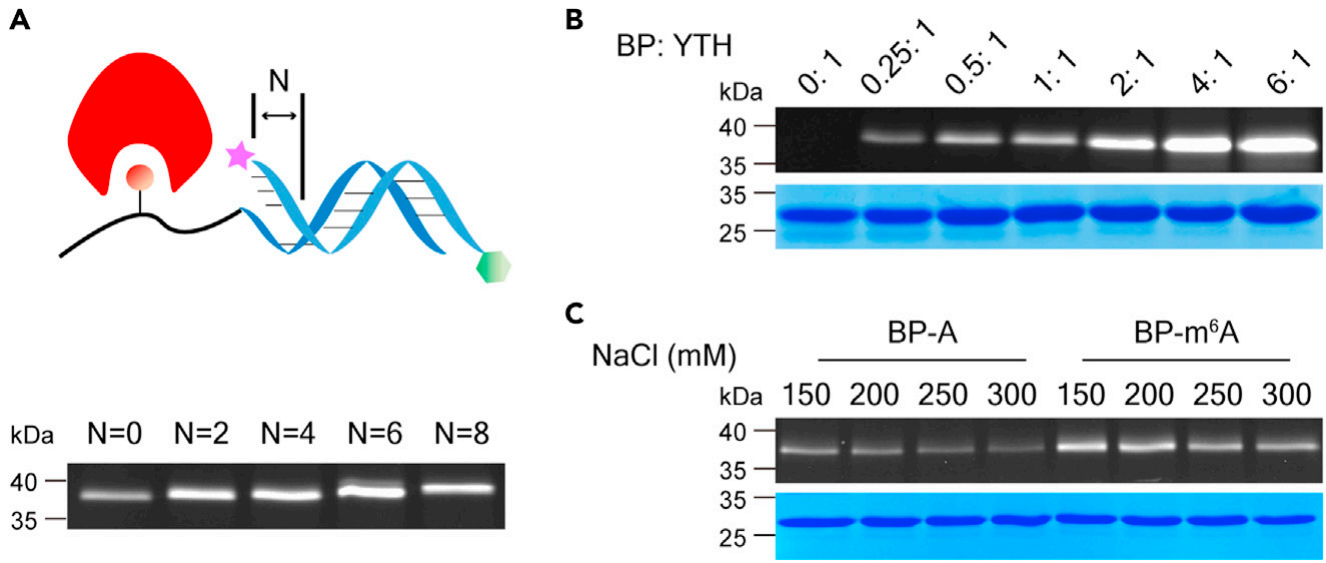
Optimization of probe and enrichment conditions (A) SDS-PAGE analysis of parallel labeling experiments using CPs containing five different N valuese. (B) The optimization of the concentration proportion of reactants. (C) The effects of various concentrations of salt ions on specific or non-specific binding.

To maximize yield of crosslinking products, a group of experiments were performed via monitoring the fluorescence intensities of the FAM-labelled targeted YTH domain. First, the spatial position of the crosslinker relative to the target was optimized by altering the chain length of the capture probe. ‘N’ value was used to represent the number of extra bases of CP compared to BP after BP/CP hybridization. To a certain extent, a larger value of N could increase accessibility of cross-linker toward its target protein. As shown in Figure 3A, N = 6 was observed to give the most efficient labeling in five different lengths. Thus, N = 6 was selected in our study. Then, the ratio of protein and probe was optimized using a gradually increased probe to a defined concentration of YTH-domain. As shown in Figure 3B, a dose-dependent increase in labeling protein was observed. Probe/protein = 4:1 has already exhibited high enrichment efficiency, a further increase did not significantly improve the yield of products and the excess probe was also increased especially at high probe concentration. Based on the above considerations, we finally chose 4:1 in the following experiments.

As reported, the nonspecific oligonucleotides-protein interaction could be suppressed by moderate amounts of salt ions (Lohman et al., 1996). So we investigated the effects of different salt concentrations on nonspecific photo-crosslinking reactions. When the salt concentration increased, the YTH-domain capturing efficiency decreased whether in specific or non-specific results (Figure 3C). Considering that the yield of specific binding products significantly decreased, we finally chose 200 mM NaCl as the reaction medium in all subsequent experiments.

### Screening m^6^A readers by DNA-guided photoactivatable RNA probe

**Figure 4:**
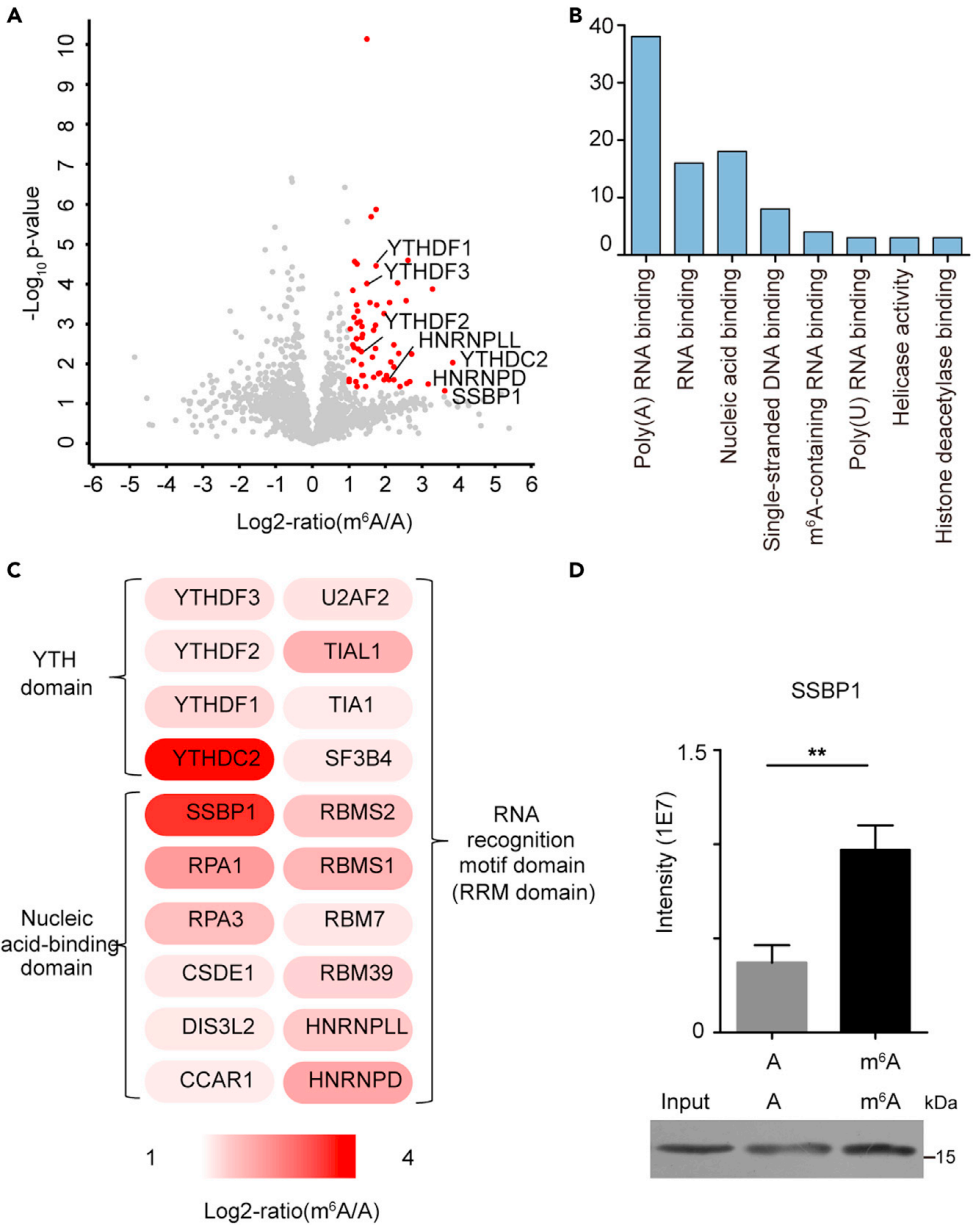
Proteomic profiling of the m^6^A interactome (A) Volcano plot of protein enrichment ratios (m^6^A/A intensity) and p values (n = 3). Red plots represent significant upregulated proteins (fold change ≥2 and p value ≤ 0.05). (B) GO enrichment analysis of m^6^A readers. (C) Protein domain enrichment analysis of m^6^A binders. (D) Streptavidin enriched protein SSBP1 detected by PRM MS analysis and western blot assay. Two-tailed Student' t-tests were used (∗∗p < 0.01).

To screen the endogenous interacting partners of m^6^A, we set up a comparative proteomics experiment by incubating the probe with the extracts of HeLa cells, with unmodified probe as control. After washes, bound proteins were subjected to on-bead trypsin digestion and subsequently analyzed by LC-MS/MS. Three independent biological replicates were performed by a DIA quantitative method (Table S5). A volcano plot was used to display the results (Figure 4A and Tables S7 and S8). We identified some known m^6^A readers, such as YTH proteins (YTHDC2 and YTHDF1-3), which showed between 2.41 and 14.37-fold preference for the m^6^A probe. Gene Ontology (GO)-term analysis of 61 significantly upregulated proteins revealed that the most enriched molecular function is poly(A) RNA binding function, as expected (Figure 4B). Furthermore, domain analysis showed that these proteins generally have YTH domain and RNA recognition motif domain (RRM), indicating that these proteins have biological correlation with mRNA at the structural level (Figure 4C). A further verification by PRM MS detection and western blot assay was shown in Figure S3, consistent with our result. Among these proteins, we found that SSBP1 has obvious affinity to m^6^A compared to unmodified RNA (ratio 12.37, Table S8), and PRM analysis and western blot assay further supported the result (Figure 4D, Table S9). Meanwhile, we noticed the downregulated proteins, including some known repelled proteins to m^6^A, such as USP10, G3BP1/2 and CAPRIN1 (Edupuganti et al., 2017). Taken together, these results confirmed the reliability of the analysis method in discovering the reader proteins of mRNA methylation.

### Screening Am readers by DNA-guided photoactivatable RNA probe

Ribose 2′-O-methylation (Nm) was recently reported as a new modification in translated regions of mRNA and plays a key function in epigenetic regulation. For example, HIV-1 increases Nm level in specific residues of the viral RNA to evade the innate immune system. Nm on snRNA is required for the fidelity of pre-mRNA splicing, and its presence in ribosomal RNA (rRNA) have impact on protein synthesis (Dai et al., 2017; Li et al., 2019; Wang et al., 2020). This modification is formed from the methylation of ribose 2′-OH moiety and can therefore occur on all 4 nucleotides. So far as we know, its binding partners remain to be explored to any of RNA types. Considering its wide distribution, we wondered whether its function is carried out by specific “readers”. However, unlike m^6^A, there is no significant consensus Nm sequence present in cells, here we chose a relatively enriched sequence NmAGAUC and 2′-O-methyladenosine (Am) to design the probe (Dai et al., 2017). And we just altered the RNA backbone of the binding probe used above without changing the capture probe, as we had optimized the chain length of it.

**Figure 5:**
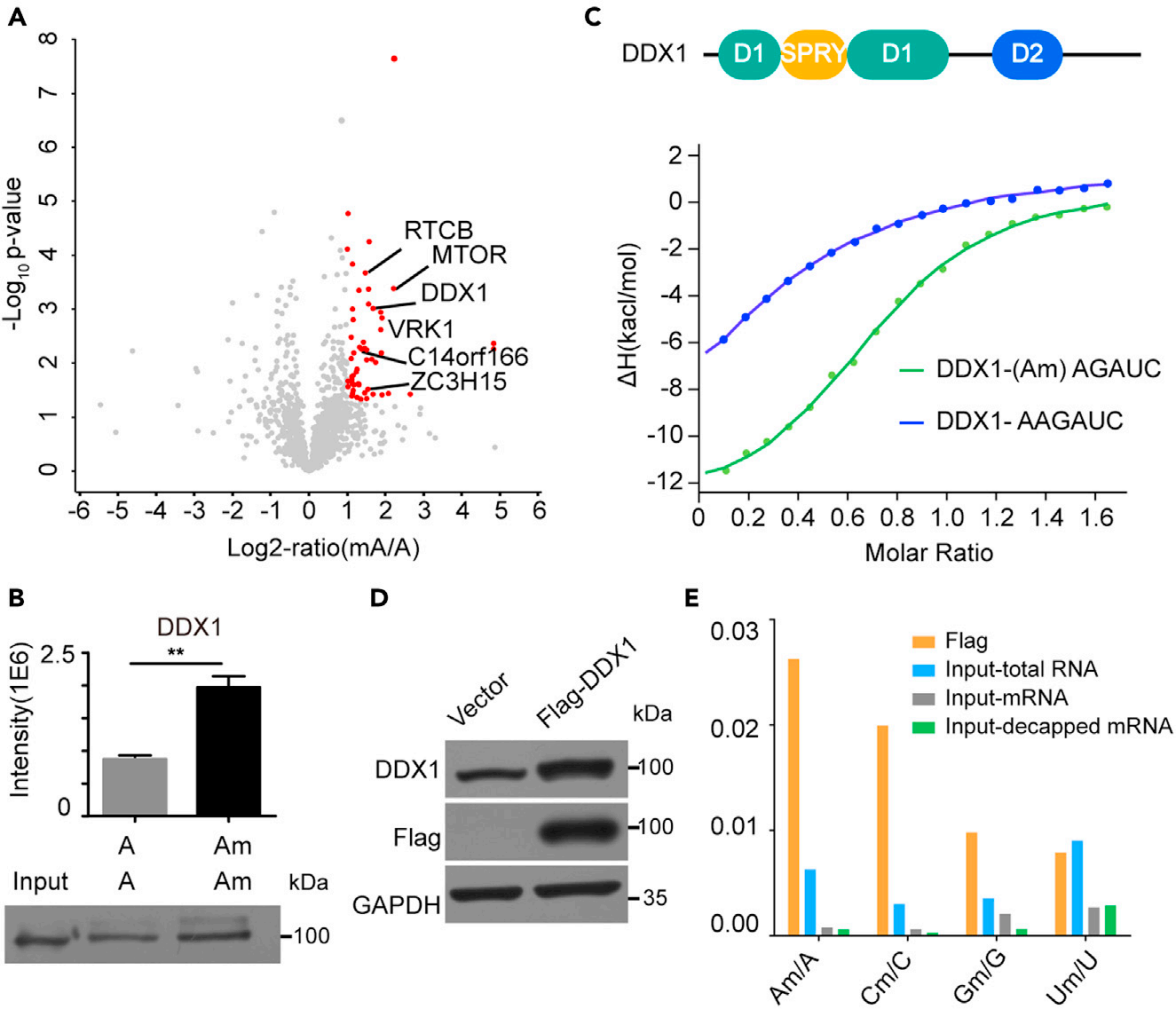
Results of Am RNA pull-down in HeLa cell lysate and selective binding of DDX1 to Am *in vitro* and *in vivo* (A) Volcano plot of protein enrichment ratios (Am/A intensity) and p-values (n = 3). Red plots represent significant upregulated proteins (fold change ≥ 2 and p-value ≤ 0.05). (B) Streptavidin enriched protein DDX1 verified by PRM analysis and western blot. Two-tailed Student' t-tests were used (∗∗p < 0.01). (C) Conserved protein domain of DDX1 and binding affinity of Am modified/un-modified ssRNA to the DDX1 measured by ITC (See also Figure S5). (D) Western blot showing overexpression of Flag-DDX1. (E). Quantification of Nm/N ratios by LC-MS/MS in Flag-immunoprecipitated RNA and input RNA.

We therefore performed a proteomic screening of the endogenous interacting partners of Am. Using the above optimized conditions, we identified 54 proteins upregulated significantly (Figure 5A, Table S10), 28 of which have ever been reported to interact with mRNA *in vivo* (Castello et al., 2012). Furthermore, GO enrichment showed that translational initiation pathway was enriched (Figure S4A), indicating that these identified proteins were potential partners with Am. For further verification, two proteins RBMX and DUS3L were selected for PRM detection and the result showed a significant 2-fold preference in binding Am containing probe over control (Figure S4B, Table S11).

Notably, a protein named DDX1 attracted our interest owing to its significant specificity to Am and structure property. The result was further validated by both PRM MS analysis and western blot assay (Figure 5B, Table S11). In addition, pull-down experiments were repeated by another BP with changed context and location of Am, illustrating that DDX1 still prefers Am (Figure S4C). To identify the major domain in DDX1 for binding Am, we constructed truncation of both domains. Isothermal titration calorimetry (ITC) assay was used for measuring the binding affinity between the domains of DDX1 and Am. As shown in Figure 5C and S5B, DDX1 showed a significantly higher binding affinity to Am modified RNA compared to unmodified RNA (Figure S1B). And deletion of D2 domain completely abolished the interaction between DDX1 and Am (Figure S5), suggesting that D2 domain is indispensable for Am recognition and binding. To evaluate in cellular binding between DDX1 and Am, we overexpressed FLAG-tagged DDX1 in HeLa cells, and used UPLC-MS/MS to assay FLAG-DDX1-immunoprecipitated RNAs (Figure 5D). The enriched RNAs were digested to nucleosides by nuclease P1 and alkaline phosphatase. As Figure 5E showed, a significant enrichment of Am was observed, indicating that DDX1 binds Am in cells.

## Discussion

In this study, we describe a method to explore interactions between RNA and reader proteins. Using photo-crosslinking, our probe is able to convert those weak and transient RNA-protein interactions into covalent ones. Taking advantage of DNA templated technique, the crosslinker is guided to the target proteins without disturbing the recognition of protein to RNA modification, thus improving the efficiency of enrichment. And therefore the DNA templated strategy could be a beneficial supplement to direct photo-crosslinking assisted pull down approach for binding proteins to mRNA modifications. In adition, in our modular probe, the probe can flexibly possess the abilities of fluorescence imaging and affinity enrichment by changing the tag carried by the CP, and also, RNA sequence on BP can be turned into other recognition modules conveniently to explore more varieties of RNA binding proteins. In addition, adding and subtracting the numbers of bases on CP enable us to advisably optimize the relative position of the crosslinker to the target proteins, which has a significant effect on the binding efficiency, avoiding complicated synthesis steps. In conjunction with mass spectrometry based proteomics, this approach can identify the transient and weak macromolecule interactions effectively, providing more insights into the revealing binding complexes of active mRNA methylation.

This approach was successfully applied to pull down known and unknown proteins that are recruited by mRNA methylation. And we found that many enriched proteins in our m^6^A pull-down experiment have overlaps with previous reports, including YTH family proteins, and some RNA binding proteins, such as SF3B4, ZCCHC8, RBM7 and HNRNPD. Here we listed parts of identified proteins that were reported previously (Table S7). Besides m^6^A binding domain YTH family proteins in our list, another group of proteins contain nucleic acid-binding domain (e.g. SSBP1, RPA1, RPA2, RPA3, CSDE1, DIS3L2, and CCAR1), which binds single-stranded nucleic acids (both RNA and DNA) and involves in multiple biological processes. For example, RPA1/2/3 and SSBP1 can bind to break DNA single strand and protect it for further repair (Richard et al., 2011). While in cellular response to double-strand breaks (DSBs), RNA moiety of DNA-RNA hybrids formed at the proximity of DSBs can be methylated, modulating the fate of the RNA and influencing the repair reaction (Xiang et al., 2017, Zhang et al., 2020). Thus it is possible that the synergy effect of proteins and modified mRNA could be enhanced by m^6^A effectors mediation. Notably, we found that SSBP1 prefers m^6^A. SSBP1 had ever been identified as a putative single-stranded DNA N6-methyldeoxyadenosine (6mA) binding protein that shows strong structural similarity with m^6^A, which implied from structure side that SSBP1 is a potential binding factor of m^6^A. In addition, we and others had identified ZCCHC8 and RBM7 as m^6^A-preferred proteins (Table S7). They belong to the nuclear RNA exosomes complex (NEXT) (Lubas et al., 2015; Wu et al., 2019), which are responsible for eliminating redundant transcripts and maintaining the steady-state levels of diverse RNA species. Previous study showed that many classes of retro-transposable elements (RTE) RNAs, particularly intronic LINE1, are strongly m^6^A-methylated in mESCs, and 43% transcripts of retrotransposon element LINE1 are found to be targeted by the ZCCHC8 and RBM7 in NEXT (Xiong et al., 2021). Thus we assume that m^6^A may act as a mark to be recognized by ZCCHC8 and RBM7 for the degradation of LINE1 RNAs. These results further illustrate that our method is useful for the profiling of binding proteins to RNA modification.

We also tried to use our method to explore binding proteins of ribose 2′-O-methylation, whose reader is unknown so far. There is no obvious consensus sequence found for Nm in RNA, whereas most RNA binding proteins have sequence selectivity to some degree, so it is a challenge for identifying Nm binding partners. Using the Am probe, we found DDX1 as a potential factor of Am modification. This physical interaction was further confirmed by western blot, ITC and RIP. Although the location and surrounding sequence of Am may influence the interaction between DDX1 and Am, for the same sequence, DDX1 still prefers Am to A (Figures 5B and S4C). DDX1 is a member of the DEAD-box protein family, which functions primarily as RNA-dependent ATP-driven RNA helicases (Kellner et al., 2015) plays an important role in RNA processing (Linder and Jankowsky, 2011). Most DEAD box family proteins have been reported to share a highly conserved helicase core that consists of two RecA-like domains (abbreviated D1 and D2), while DDX1 is unique amongst other family members since it contains a SPRY domain insertion. SPRY domain has a conserved patch of positive charge surface, which may be important for the specific interaction of DDX1 and negative RNA skeleton (Kellner and Meinhart, 2015). The RNA helicases have been thought to be ATP-driven switches for modulating RNA secondary structure, which functions in a similar way with methylation that can change the conformation of RNA, we hypothesized that they might regulate synergistically the rearranging of RNA-protein complexes and further influence the downstream functions of RNA. As we have demonstrated the binding preference for DDX1 to Am than A, biological studies could be taken to further explore the regulation of DDX1 to site-specific Am modification in different types of RNA molecules.

In summary, we have developed an efficient probe-based chemical proteomics for profiling the interactome of mRNA methylation. We also identified new binding partners of m^6^A, expanding the catalog of m^6^A interactome. A potential Am binding factor, DDX1, was further uncovered to specifically recognize Am, providing clues for related regulation mechanism. Our work provides a tool for interrogating the interactome of RNA methylation, which holds a great potential for a broad and general application in discovering the binding proteins of any RNA modifications, further to reveal the molecular mechanisms of the RNA modification-mediated post-transcriptional gene regulation.

### Limitations of the study

This study describes a probe-based chemical proteomics for profiling the interactome of mRNA methylation. We introduce a DNA templated technique so that the crosslinker can be guided to the target proteins without disturbing the recognition of protein to RNA modification. However, to avoid stability secondary structure formation and to reduce interference from endogenous DNA, a careful design of oligonucleotide sequence in probes is needed. Moreover, this approach was applied in HeLa lysate to pull down known and unknown proteins that are recruited by RNA methylation, to determine whether our results are universally applicable, further studies using different cell lines should be done.

## STAR★Methods

### Key resources table

**Table:** 

REAGENT or RESOURCE	SOURCE	IDENTIFIER
**Antibodies**		

Rabbit polyclonal anti- SSBP1	ABclonal	Cat#A6987; RRID:AB_2767543
Rabbit polyclonal anti- DDX1	ABclonal	Cat#A6575; RRID:AB_2767169
Rabbit polyclonal anti- ZCCHC8	ABclonal	Cat#A9563; RRID:AB_2772946
Rabbit polyclonal anti- RBMS1	ABclonal	Cat#A3079; RRID:AB_2764882

**Chemicals, peptides, and recombinant proteins**		

Bovine serum albumin	Sigma−Aldrich	Cat#9048-46-8
SDA (NHS-Diazirine) (succinimidyl 4,4′-azipentanoate)	Thermo Fisher Scientific	Cat#26167
Streptavidin Agarose	Thermo Fisher Scientific	Cat#20349
Nuclease P1	Sigma−Aldrich	Cat#N8630
Alkaline Phosphatase (Shrimp)	Solelybio	Cat#2660A
RNA 5' Pyrophosphohydrolase (RppH)	NEW ENGLAND BioLabs	Cat#M0356S
Recombinant DNase I (RNase-free)	Takara	Cat#2270A

**Critical commercial assays**		

EZ-Magna RIP Kit	Millipore	Cat#17-701
Dynabeads mRNA Purification Kit	Thermo Fisher Scientific	Cat#61006
Pierce™ BCA Protein Assay Kit	Thermo Fisher Scientific	Cat#23227

**Deposited data**		

MS/MS analysis for protein quantification	This paper	Table S8. Endogenous m6A binders in three biological replicates using DIA approach, related to Figure 4, Table S9. Verification of m6A binders in two biological replicates using PRM approach, related to Figure 4, Table S10. Endogenous Am binders in three biological replicates using DIA approach, related to Figure 5, Table S11. Verification of Am binders in two biological replicates using PRM approach, related to Figure 5; ProteomeXchange Dataset identifier: PXD019480

**Experimental models: Cell lines**		

HeLa CCL-2	ATCC	Cat#CCL-2; RRID:CVCL_0030
Oligonucleotides		
Primer for pET28a-YTHDF2 (See Table S1)	This paper	N/A
Primer for pcDNA 3.1-flag-DDX1 (See Table S1)	This paper	N/A
Primer for pET28a-DDX1 and truncation (See Table S1)	This paper	N/A
Binding probes and Capture probes (See Table S2)	This paper	N/A

**Recombinant DNA**		

Plasmid: pET28a-YTHDF2(396-580)	This paper	N/A
Plasmid: pET28a-DDX1 (1-728)	This paper	N/A
Plasmid: pcDNA 3.1-flag-DDX1	This paper	N/A

### Resource availability

#### Lead contact

Further information and requests for resources and reagents should be directed to and will be fulfilled by the Lead Contact, Kai Zhang (kzhang@tmu.edu.cn).

#### Materials availability

This study did not generate new unique reagents.

### Experimental model and subject details

#### Cell lines

HeLa cells are cultured at 37°C in Dulbecco's modified Eagle medium (DMEM; Biological Industries, Israel) supplemented with 10% fetal bovine serum (Biological Industries, South American origin) in a humidified incubator with 5% CO2.

### Method details

#### Chemical synthesis and characterization of synthetic probe

Capture probe is synthesized via the rapid substitution reaction between amino group and NHS-ester-conjugated diazirine (Scheme S1). Amino modified DNA was centrifuged for 5 min at a speed of 10,000 g before it was dissolved in NaHCO_3_ buffer (0.1 M, pH 8.5) to a concentration of 100 μM. Then, succinimidyl-ester diazirine (SDA) dissolved in DMSO was added to the DNA solution along with 5 equiv. Finally, the mixture is thoroughly mixed and shaken at 25°C for 2 h. The products were purified via ethanol precipitation. Supernatant was removed and precipitated pellet was washed three times with ethanol and re-dissolved in pure water. Oligonucleotides were quantified by a NanoDrop 2000C (Thermo Fisher Scientific). All the oligonucleotides were characterized by ESI-MS (LCQ DECA XP, Thermo Fisher Scientific). Oligonucleotides are analyzed in negative ion mode. The sequence and spectral data are shown in Table S2 and Figure S2.

#### Expression, purification and characterization of recombinant proteins

Plasmids encoding cDNA were obtained from Jiahuai Han's Lab: YTHDF2 (13328) and DDX1 (7395). Sequences were cloned into pET28a vector for protein expression (YTHDF2: EcoR I, Hand III; DDX1: BamHI, XhoI) (See Table S1 for primers cloning pET28a-YTHDF2, pET28a-DDX1 and DDX1 truncations). YTHDF2-YTH (396-580) was expressed for 20 hours at 16°C with 0.8 mM IPTG in Escherichia coli strain BL21 (DE3), DDX1 and truncations was expressed for 16 hours at 18°C with 0.5 mM IPTG in Escherichia coli strain Rosetta (DE3). Cells were lysed by sonication and the protein was purified using Ni-NTA affinity resin (20349, Thermo Fisher Scientific) according to the manufacturer's recommendations. The purity of recombinant proteins exceeded 90% as detected SDS-PAGE analysis (Figure S1). Protein concentrations were determined by BCA Protein Assay Kit (23227, Thermo Fisher Scientific) and characterized by LC-MS/MS. Mass spectrometric data of the recombinant proteins are shown in Figure S1.

#### Fluorescence labeling of YTH domain

BP and YTH-domain or YTH-domain contaminated by BSA or cell lysates were first mixed in reaction buffer (20 mM Tris pH 7.5, 200 mM NaCl buffer) on ice for 1 h. Then fluorescein labelled CP was added and the mixture was incubated on ice for another 1 h. After irradiation for 6 min on ice using UVP CL-1000 at 365 nm, 5×loading buffer was added to the sample and boiled for 10 min at 95°C, the protein sample is separated by 12% SDS−PAGE and further analyzed by scanning the gels using a bio-ray imager with 365 nm excitation.

#### Affinity pull-down experiment to identify RNA binding proteins in cell extracts

The extracts of HeLa cells were obtained using RIPA Lysis Buffer (P0013D, Beyotime) and concentration of protein samples was measured by BCA assay.Briefly, CP (200 μM, 20 μl) was first mix with 40 μL of pre-washed streptavidin-bound beads and incubated with rotation for 2 h at 4°C. Cell lysates corresponding to 6 mg of protein and BP (200 μM, 20 μl) were added and rotated at 4°C for another 2 hours followed by the UV irradiation that performed on ice for about 6 min. Then precipitation is washed for three times by wash buffer 1 (2 M NaCl, 1 mM EDTA, 10 mM HEPES, pH 7.4, 0.01% Triton X100) two times and wash buffer 2 (2 M NaCl, 1 mM EDTA, 10 mM HEPES, pH 7.4, 0.1% Triton X100) one time to remove the non-specific protein. Finally, the resins were boiled at 95°C for 10 min. Centrifuge for 1 min at 500 g for 1 min, the supernatant is loaded onto the gels to be separated by SDS-PAGE and analyzed by western blot. Or the captured proteins were digested on beads with trypsin and then analyzed by LC-MS/MS.

#### Mass spectrometry analysis of the protein samples

MS/MS analysis for protein quantification in a data independent acquisition (DIA) mode. For protein quantification, we used a DIA quantitation method by following a previously report (Bruderer et al., 2015) with some modifications. Briefly, in the generation of the DIA database, we performed the DDA method, and the offline-HPLC were used to separate 10 μg enzymatic peptides of the whole protein lysate of HeLa cells. In brief, the enzymatic peptides were loaded into an Xbridge-Peptide BEH C18 column (Waters), the gradient elution was performed from 0 to 8% buffer B (95% ACN, 5% H_2_O pH 8.0) in 3 min, 8-18% buffer B in 26 min, 18-32% buffer B in 30 min, 32-95% buffer B in 1 min, at a flow rate of 1.0 ml/min. And the samples were merged into 10 fractions during the process of drying. Then the 10 fractions were desalted by using C18 Stage-tips, and analyzed by an Easy-nLC 1200 connected to a Q Exactive Plus mass spectrometer (Thermo Fisher Scientific). Two technical replicates of MS were performed for each fraction. The peptides were separated by a 140 min linear gradient (3.2%–8% ACN with 0.1% formic acid at 300 nl/min in 2 min, followed by a linear increase to 28.8% ACN in 120 min and 80% in 5 min). Notably, this gradient was also applied to DIA and PRM methods. In the DDA mode of the MS, the full scan was performed between 400–1,200 m/z. The automatic gain control (AGC) target for the MS/MS scan was set to 5e4. Collision energy was set to 27%, and the loop count was 15.

The DIA-MS method consisted of a MS1 scan from 400 to 1200 m/z (AGC target of 5e6 or 120 ms injection time). Then, 19 DIA windows were acquired at 35,000 resolution (AGC target 3e6 and auto for injection time) (Table S3). Stepped collision energy was10% at 25%. For the target proteins validation by using PRM method, we used the same settings of the full MS scan in the DIA method. And in the PRM MS/MS spectra, the resolution was 17500, the AGC target was set to 1e5, and the isolation window was set to 2.0 m/z, collision energy was set to 27%. And the target peptides information was showed in Table S4.

For generation of the spectral libraries, 20 DDA measurements described above was used. And a spectral library was generated using the function “spectral library generation” in the software Spectronaut Pulsar X , a mass spectrometer software from Biognosys. The DDA files were searched against the human UniProt fasta database (20217 entries). Besides annotation of precursors and fragment ions, the peptides of indexed Retention Time (iRT) kit were also contained in the library.

Also the DIA data and the PRM data were analyzed by Spectronaut Pulsar X with default. MS raw data were searched against the DIA database described above. The DIA quantitation were performed three biological replicates, and the PRM validate experiments were performed two biological replicates and a high Pearson correlation was found in these replicates (Tables S5–S6). Statistical analysis of target proteins was performed by Student's unpaired t test using GraphPad InStat software. P<0.05 was considered to be statistically significant.

#### Western blot analysis

The protein samples were separated on a 12% polyacrylamide SDS gel and transferred to a nitrocellulose membrane (Pall Corporation, 0.22 μm). The membranes were incubated with primary antibodies overnight at 4°C. After washing, the membranes were incubated with goat anti-mouse HRP-conjugated secondary antibody (1:10000) or goat anti-rabbit HRP-conjugated secondary antibody (1:10000), for 2 h at room temperature.

#### Isothermal titration calorimetry measurements

All ITC measurements were recorded at 25°C using a Malvern MicroCal PEAQ-ITC (MicroCal). And RNAs used for ITC binding experiments were purchased from GenScript Biotech Corp (Nanjing, China). The purity of all purchased RNAs was >90%. All proteins and RNAs are dissolved in the same buffer containing 20 mM Tris, pH 7.5, 150 mM NaCl before use. 19 injections were performed by injecting 2 μl 400–700 μM RNAs into a sample well containing 50–80 μM of proteins. The concentrations of the proteins were determined by BCA Assay. The concentration of RNAs were estimated with absorbance spectroscopy using the extinction coefficients OD 260nm. Binding isotherms were analyzed and fitted in a one-site binding model by Malvern MicroCal PEAQ-ITC Analysis Software (MicroCal).

#### Plasmid construction for RNA binding protein immunoprecipitation (RIP)

Plasmids with high purity for mammalian cell transfection were prepared with EndoFree Mini Plasmid Kit (DP118-02, TIANGEN). (See Table S1 for Primers cloning pcDNA 3.1-flag-DDX1) Transfection was achieved using Lipofectamine 2000 Transfection Reagent (11668500, Invitrogen) according to the manufacturer's protocol.

RIP was performed using the EZ-Magna RIP Kit (17-701, Millipore) according to the manufacturer's instructions with some modifications. Briefly, three 15 cm dishes of confluent HeLa cells transiently overexpressing FLAG-tagged DDX1 were harvested by scrape. 5μg of FLAG (M185-3L, MBL) was conjugated to 50 ul protein A/G magnetic beads by incubation for 4 h at 4°C, followed by washing three times and incubation with HeLa cells extraction in RIP lysis buffer at 4°C overnight. After washing for five times, beads and input were resuspended in RIP wash buffer, followed by DNase I (2270A, Takara) digestion at 37°C for 15 min and incubation with 50μg of proteinase K at 55°C for 30 min. Input and co-immunoprecipitated total RNAs were recovered by TRIzol. The total RNA of input was defined as “input-total RNA”, furthermore, mRNA enriched from input-total RNA by Dynabeads mRNA Purification Kit (61006, Thermo) were defined as “input-mRNA”, additionally, input-mRNA decapped by RNA 5' Pyrophosphohydrolase (M0356S, NEB) were defined as “input-decapped mRNA”. The extracted RNAs were analyzed by UPLC-MS/MS.

#### UPLC-MS/MS analysis

5ug RNA of each sample were digested by nuclease P1 (N8630, Sigma) in 25 μl of buffer containing 10 mM sodium acetate buffer (pH 5.3), 2.5 mM zinc chloride, and 25 mM sodium chloride at 42°C for 2 h, followed by the addition of alkaline phosphatase (2660A, Solelybio). After an additional incubation at 37°C for 2 h, the sample was dried up, resuspended in Buffer A, and injected into LC-MS/MS. Nucleosides were separated by Waters ACQUITY UPLCTM HSS T3 column (2.1 × 100 mm, 1.7 m) with on-line mass spectrometry detection using an Agilent 6600 QQQ triple-quadrupole LC mass spectrometer in positive electrospray ionization mode. The assay was completed at a flow rate of 0.2 ml/min and column temperature of 25°C. Mobile phases included RNase-free water containing 0.01% formic acid (Buffer A) and 80% Methanol in aqueous 0.01% formic acid (Buffer B). A 40 min gradient was developed to obtain optimum separation of modified nucleosides. The nucleosides were quantified by using retention time and the nucleoside to base ion mass transitions of 298–152 (Gm), 282.1–136.1 (Am), 259–113 (Um), 258–112.1 (Cm), 284–152 (G), 268–136 (A), 245–113.1(U) and 244–112 (C). The Nm level was calculated as the peak area ratio of Nm to N.

### Quantification and statistical analysis

#### Quantitative analysis of mass spectrometry data

Quantitative analysis of significant differences and data visualization was performed using the Perseus software platform (Tyanova et al., 2016) (version 1.5.1.6) using PG.Quantity (reported from the software Spectronaut Pulsar X) of biological replicates. A volcano plot was generated based on PG.Quantity applying F-test and two-way Student's t test to probe for significant difference of protein abundance between the modified and un-modified RNA pull-down groups (Figures 4A and 5A).

## Data Availability

The datasets generated during this study are available publicly at ProteomeXchange (http://proteomecentral.proteomexchange.org). Dataset identifier: PXD019480. This paper does not report original code. Any additional information required to reanalyze the data reported in this paper is available from the lead contact upon request.

## Supplemental information

Document S1. Scheme S1, Figures S1–S5, and Tables S1–S7

Table S8. Endogenous m^6^A binders in three biological replicates using DIA approach, related to Figure 4

Table S9. Verification of m^6^A binders in two biological replicates using PRM approach, related to Figure 4

Table S10. Endogenous Am binders in three biological replicates using DIA approach, related to Figure 5

Table S11. Verification of Am binders in two biological replicates using PRM approach, related to Figure 5
