# Supplementary material for: DNA-guided photoactivatable probe-based chemical proteomics reveals the reader protein of mRNA methylation
Source: iScience. 2021 Aug 28;24(9):103046. doi: 10.1016/j.isci.2021.103046 (PMC8441146; doi:10.1016/j.isci.2021.103046)
Supplement: Document S1. Scheme S1, Figures S1–S5, and Tables S1–S7 [file mmc1.pdf]

iScience, Volume *24*

Supplemental information

DNA-guided photoactivatable probe-based chemical proteomics reveals the reader protein of mRNA methylation

Yepei Huang, Xue Bai, Zhenchang Guo, Hanyang Dong, Yun Fu, Hui Zhang, Guijin Zhai, Shanshan Tian, Ye Wang, and Kai Zhang

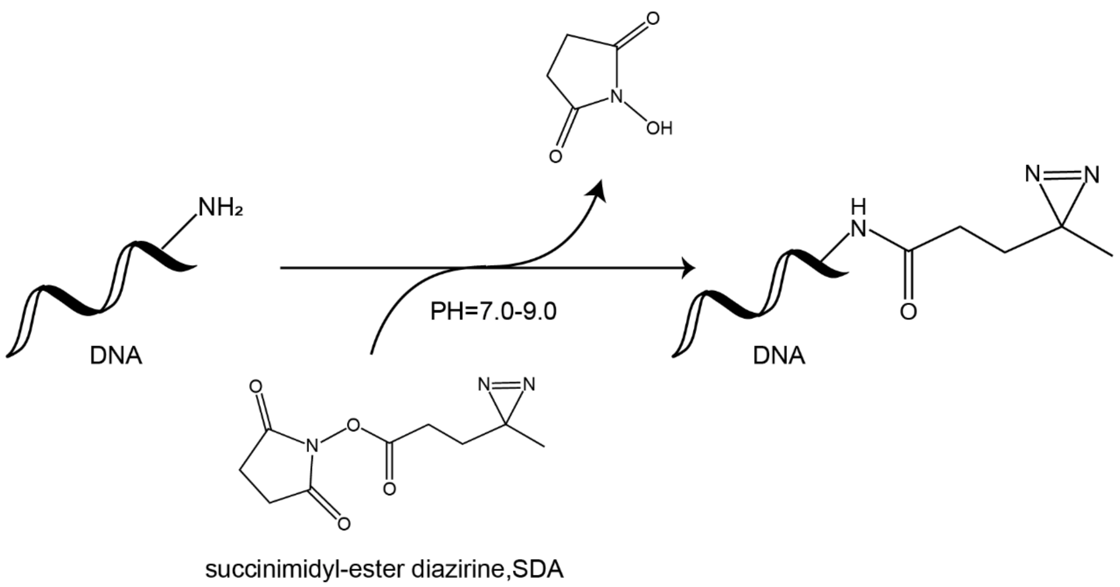

Scheme 1 Synthesis of capture probe using amino modified DNA and succinimidyl-ester diazirine (SDA). Related to STAR Methods.

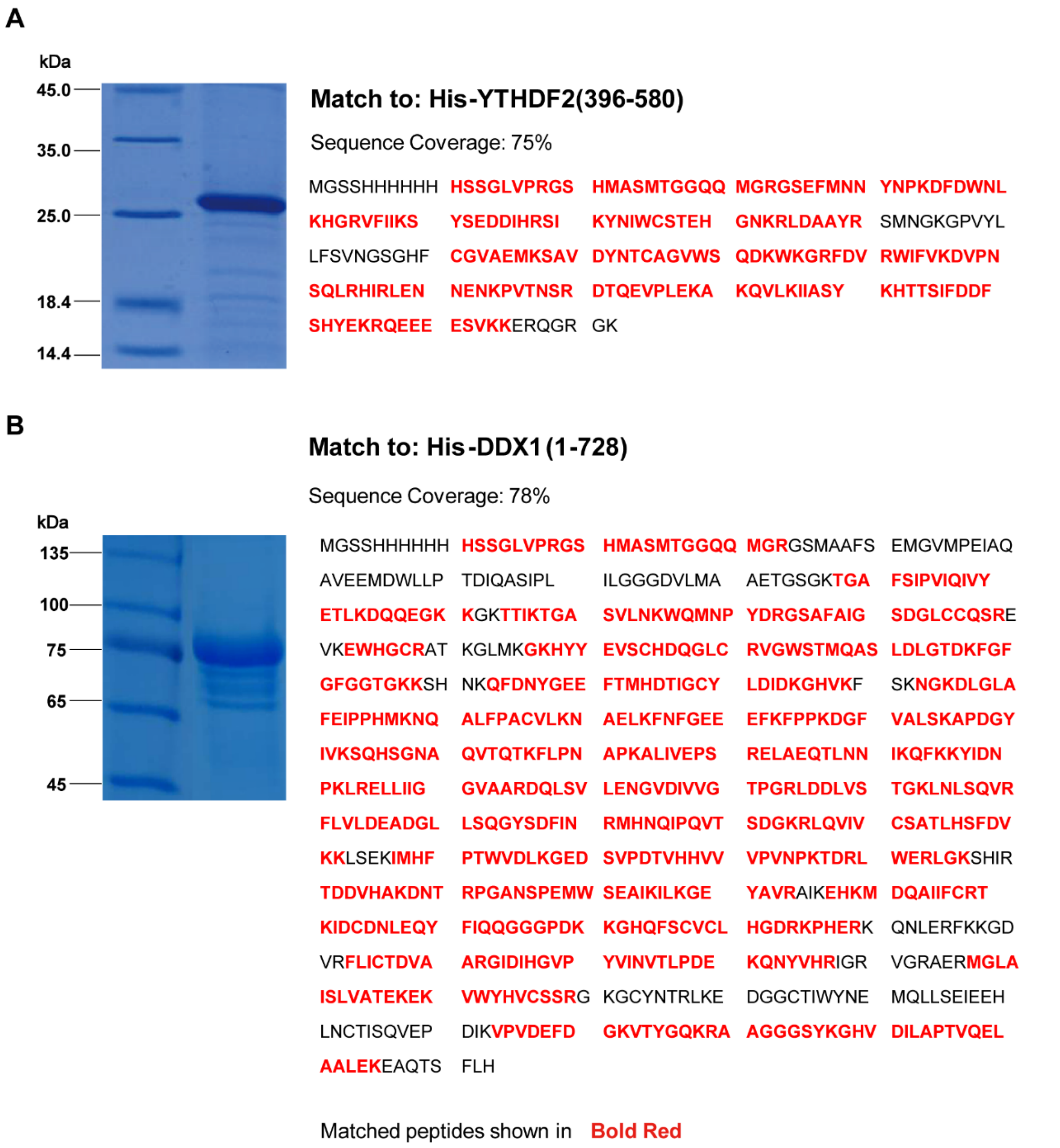

Figure S1. The MS characterization of purified proteins. Related to STAR Methods. (A) SDS-PAGE and The coverage rate of YTHDF2-YTH identified by LC-MS/MS matched to the database. (B) SDS-PAGE and The coverage rate of DDX1 identified by LC-MS/MS matched to the database.

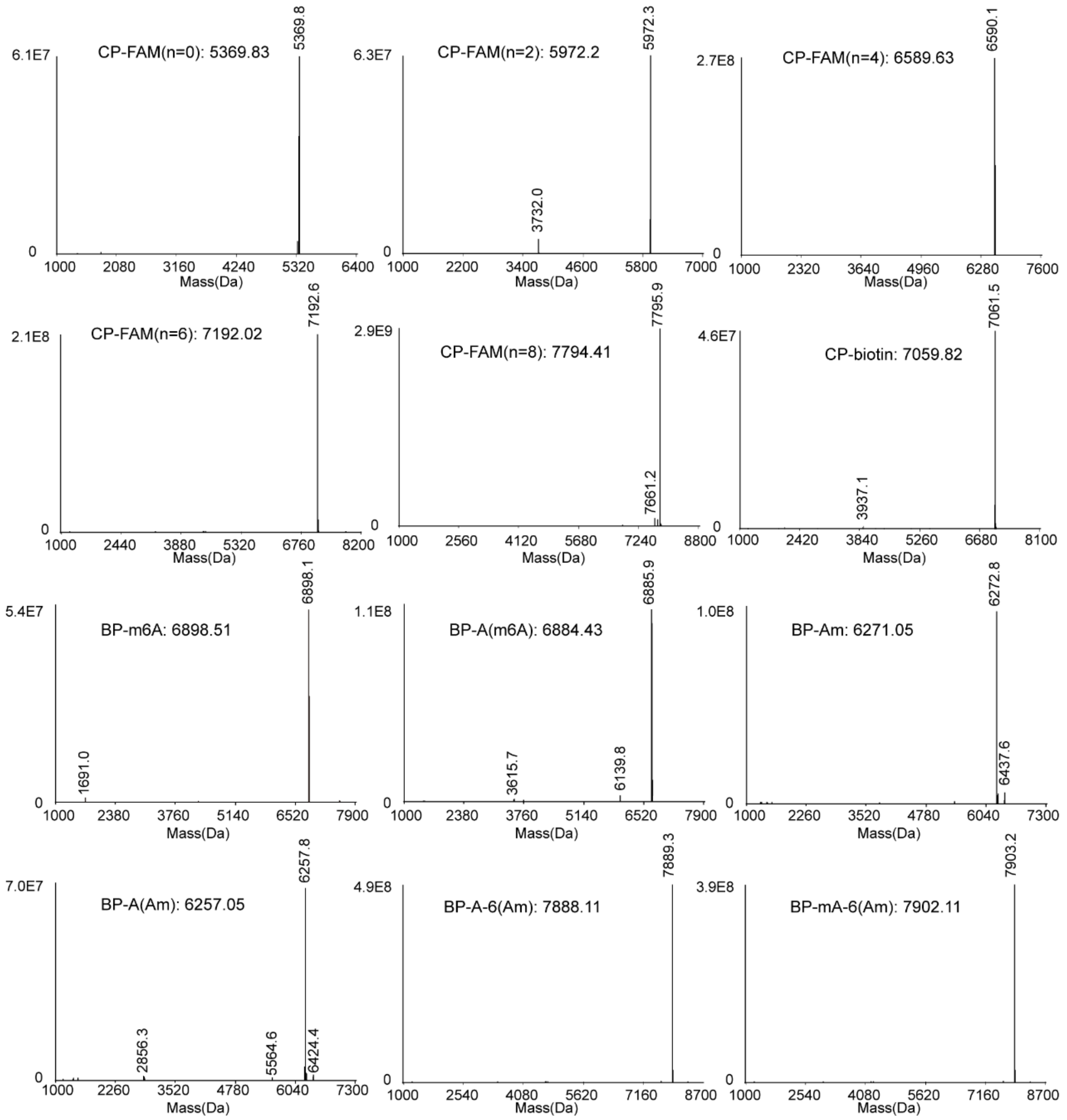

Figure S2. MS characterization of the binding probes and DNA-based capture probes. Related to STAR Methods. Theoretical molecular weight of every probe has been shown in top right corner of their spectrogram.

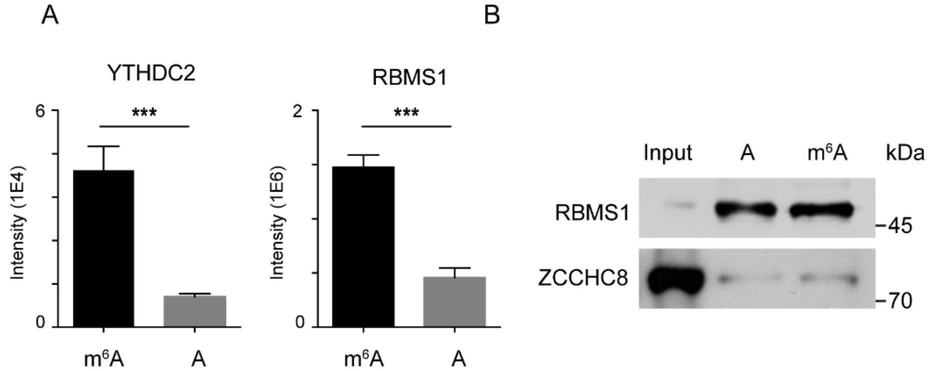

Figure S3. Detection of m^{6}A binders. Related to Figure 4. (A) Histogram of two target protein YTHDC2 and RBMS1 verified by PRM scan mode. (B) Target protein RBMS1 and ZCCHC8 verified by western blot.

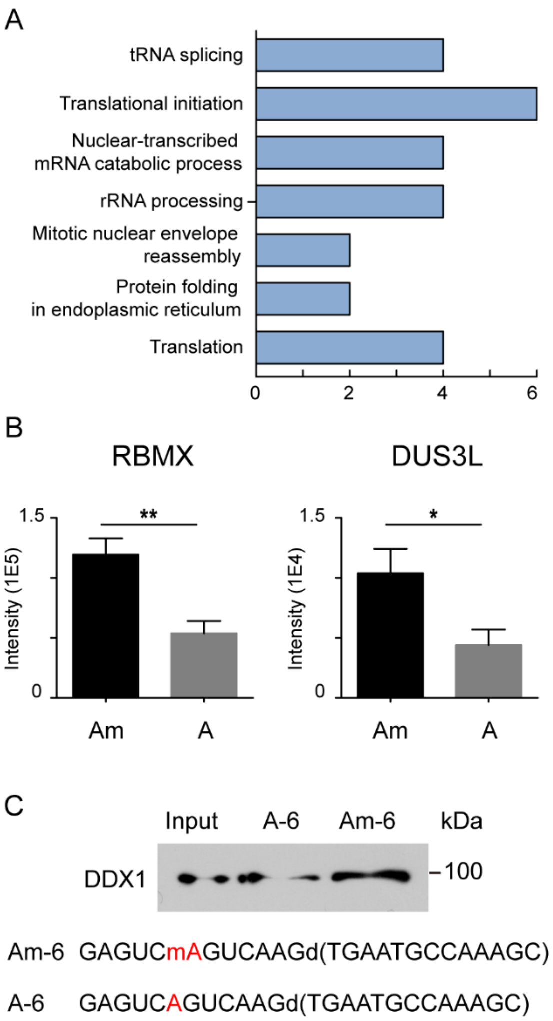

Figure S4. Analysis of Am binders. Related to Figure 5. (A).GO enrichment analysis of Am binding proteins. (B) Histogram of two target protein DUS3L and RBMX verified by PRM scan mode.(C) Streptavidin enriched protein DDX1 verified by western blot with another BP (shown below).

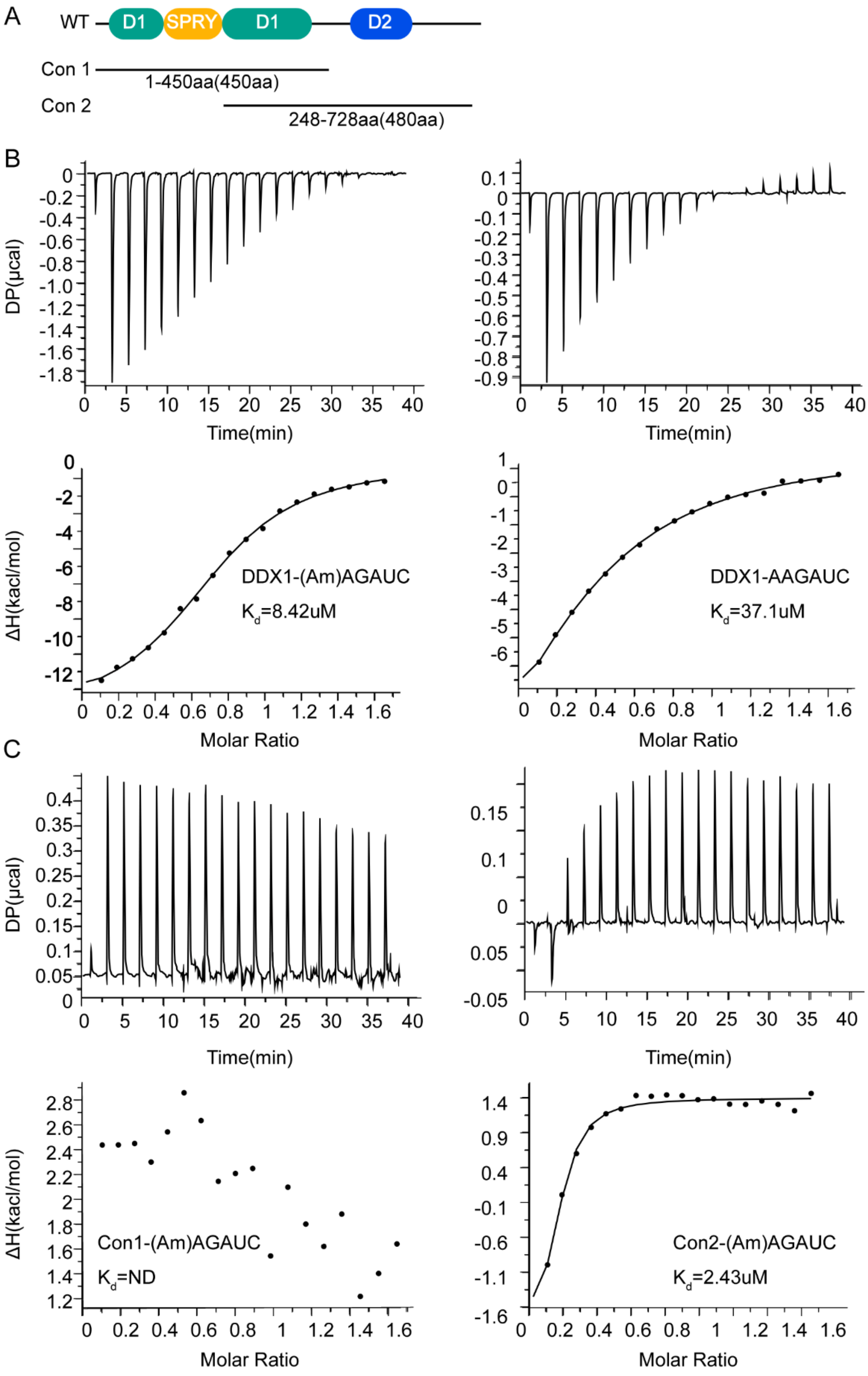

Figure S5. In vitro verification of DDX1 for Am binding. Related to Figure 5. (A). Schematic structures showing a summary of DDX1 trucations used in this study. (B). ITC showed in vitro binding affinity of modified/unmodified single-stranded RNA (ssRNA) with wild-type DDX1. (C). ITC showed in vitro binding affinity of modified ssRNA with DDX1 truncations.

Table S1. Primer used in cloning. Related to STAR Methods.

Recombinant DNA	Forward (F) or Reverse (R)	Sequence
pET28a-YTHDF2	F	CCGGAATTCATGTCGGCCAGCAGCCTCTT
	R	CCCAAGCTTTTATTTCCCACGACC
pET28a-DDX1	F	GCGGGATCCATGGCGGCCTTCTCC
	R	CCGCTCGAGTCAATGCAGGAAAGATG
pET28a-DDX1-con1	F	GCGGGATCCATGGCGGCCTTCTCC
	R	CCGCTCGAGGGGATTTACTGGGACAACAACATGGTGTACAG
pET28a-DDX1-con2	F	GCGGGATCCAAGTTTCCACCAAAAGATGGCTTTGTTGC
	R	CCGCTCGAGTCAATGCAGGAAAGATG
pcDNA 3.1-flag-DDX1	F	CGCGGATCCATGGACTACAAGGACGACGATGACAAGGCGGCCTTCTCCGAGATGGGTGTAATGCCTGAGA
	R	CGCGGATCCATGGACTACAAGGACGACGATGACAAGGCGGCCTTCTCCGAGATGGGTGTAATGCCTGAGA

Table S2. Oligonucleotides used in this study. Related to STAR Methods.

Name	Sequence
BP-m^6A	5’-r[GG(m^6A)CUGUAC]d[TGAATGCCAAAGC]-3’
BP-A(m^6A)	5’-r[GGACUGUAC]d[TGAATGCCAAAGC]-3’
BP-Am	5'-r[mAAGAUCG]d[TGAATGCCAAAGC]-3'
BP-A(Am)	5'-r[AAGAUCG]d[TGAATGCCAAAGC]-3'
BP-Am-6	5'-r[GAGUCmAGUCAAG]d[TGAATGCCAAAGC]-3'
BP-A-6(Am)	5'-r[GAGUCAGUCAAG]d[TGAATGCCAAAGC]-3'
CP-FAM-DNA (N=0)	5’-Carboxy-fluorescein-ACGCTTTGGCATTCA-C6NH2-3’
CP-FAM-DNA (N=2)	5’-Carboxy-fluorescein-ACGCTTTGGCATTCACA-C6NH2-3’
CP-FAM-DNA (N=4)	5’-Carboxy-fluorescein-ACGCTTTGGCATTCACATA-C6NH2-3’
CP-FAM-DNA (N=6)	5’-Carboxy-fluorescein-ACGCTTTGGCATTCACATACA-C6NH2-3’
CP-FAM-DNA (N=8)	5’-Carboxy-fluorescein-ACGCTTTGGCATTCACATACAAC-C6NH2-3’
CP-biotin-DNA (N=6)	5’-biotin- ACGCTTTGGCATTCACATACA-C6NH2-3’

Table S3. DIA isolation windows. Related to STAR Methods.

Window Nr.	Range	Window size
1	400-430	30
2	428-459	31
3	457-483	26
4	481-506	25
5	504-531	27
6	529-554	25
7	552-576	24
8	574-600	26
9	598-624	26
10	622-650	28
11	648-676	28
12	674-704	30
13	702-735	33
14	733-771	38
15	769-810	41
16	808-856	48
17	854-914	60
18	912-1000	88
19	998-1200	202

Table S4. PRM target peptides information. Related to STAR Methods.

Protein	Peptide	m/z (Da)	Charge	MH+ (Da)
SSBP1	QATTIIADNIIFLSDQTK	996.53394	2	1992.0606
SSBP1	SGDSEVYQLGDVSQK	806.37616	2	1611.745
YTHDC2	SQDWGSAGLGGVFK	704.84259	2	1408.6779
YTHDC2	IGQTIGYQIR	574.82214	2	1148.637
RBMS1	TPPGVSAPTEPLLCK	783.91064	2	1566.8140
RBMS1	GLPPHTTDQDLVK	710.87354	2	1420.7397
DDX1	DQLSVLENGVDIVVGTPGR	984.5845	2	1969.1690
DDX1	GIDIHGVPYVINVTLPDEK	1040.1760	2	2079.3521
RBMX	VEQATKPSFESGR	718.3597	2	1435.712
RBMX	IVEVLLMK	472.7951	2	944.5829
DUS3L	DFTNYGLEHWGSDTQGVEK	1091.982	2	2182.957
DUS3L	FSQGPTPAAAVPEGTAAEGAPR	1041.512	2	2082.017
iRT2	GAGSSEPVTGLDAK	644.819	2	
iRT1	LGGNEQVTR	487.2531	2	

Table S5. The Pearson correlation in DIA quantitation replicates of m^6A pull-down experiment. Related to Figure 4.

Name	m^6A-1-1	m^6A-1-2	m^6A-1-3	m^6A-2-1	m^6A-2-2	m^6A-2-3	m^6A-3-1	m^6A-3-2	m^6A-3-3
m^6A-1-1	NaN	0.9996	0.9473	0.7338	0.7873	0.7830	0.9955	0.9632	0.9943
m^6A-1-2	0.9996	NaN	0.9402	0.7267	0.7803	0.7742	0.9934	0.9566	0.9937
m^6A-1-3	0.9473	0.9402	NaN	0.7988	0.8554	0.8648	0.9671	0.9962	0.9489
m^6A-2-1	0.7338	0.7267	0.7988	NaN	0.9905	0.9786	0.7639	0.7874	0.7213
m^6A-2-2	0.7873	0.7803	0.8554	0.9905	NaN	0.9889	0.8176	0.8427	0.7759
m^6A-2-3	0.7830	0.7742	0.8648	0.9786	0.9889	NaN	0.8199	0.8495	0.7634
m^6A-3-1	0.9955	0.9934	0.9671	0.7639	0.8176	0.8199	NaN	0.9783	0.9890
m^6A-3-2	0.9632	0.9566	0.9962	0.7874	0.8427	0.8495	0.9783	NaN	0.9666
m^6A-3-3	0.9943	0.9937	0.9489	0.7213	0.7759	0.7634	0.9890	0.9666	NaN

Table S6. The Pearson correlation in DIA quantitation replicates of Am pull-down experiment. Related to Figure 5.

Name	Am-1-1	Am-1-2	Am-1-3	Am-2-1	Am-2-2	Am-2-3	Am-3-1	Am-3-2	Am-3-3
Am-1-1	NaN	0.9987	0.9976	0.9490	0.9464	0.9437	0.9836	0.9863	0.9868
Am-1-2	0.9987	NaN	0.9987	0.9437	0.9428	0.9400	0.9784	0.9801	0.9816
Am-1-3	0.9976	0.9987	NaN	0.9375	0.9371	0.9326	0.9741	0.9790	0.9804
Am-2-1	0.9490	0.9437	0.9375	NaN	0.9979	0.9980	0.9333	0.9380	0.9332
Am-2-2	0.9464	0.9428	0.9371	0.9979	NaN	0.9991	0.9237	0.9292	0.9250
Am-2-3	0.9437	0.9400	0.9326	0.9980	0.9991	NaN	0.9236	0.9264	0.9225
Am-3-1	0.9836	0.9784	0.9741	0.9333	0.9237	0.9236	NaN	0.9966	0.9974
Am-3-2	0.9863	0.9801	0.9790	0.9380	0.9292	0.9264	0.9966	NaN	0.9992
Am-3-3	0.9868	0.9816	0.9804	0.9332	0.9250	0.9225	0.9974	0.9992	NaN

Table S7. **Proteins identified in Figure 4 that also been reported to bind m^{6}A modified RNA. Related to Figure 4.**

Identified m^{6}A binding proteins	Related publications
YTHDF1	Wang et.al *Cell* 161: 1388–1399 2015 (Systematically characterized by multiple assays)
YTHDF2	Wang et.al *Nature* 505: 117 2014 (Systematically characterized by multiple assays)
YTHDF3	Li et.al *Cell Res* 27: 444 2017 (Systematically characterized by multiple assays)
YTHDC2	Hsu et.al *Cell Res* 27: 1115 2017 (Systematically characterized by multiple assays)
CSDE1	Arguello et.al *J Am Chem Soc* 139: 17249-17252 2017 (Bind m6A probe in HeLa lysate)
CAMK2G	Edupuganti et.al Nat Struct Mol Biol 24:870-878 2017 (Bind m^{6}A probe in mNPC cytoplasm)
HNRNPD	Song et.al Autophagy15(8):1419-1437 2019 (Bind m^{6}A modified TFEB pre-mRNA)
PATL1	Edupuganti et.al Nat Struct Mol Biol 24:870-878 2017 (Bind m^{6}A probe in HeLa cytoplasm)
RBM7	Edupuganti et.al Nat Struct Mol Biol 24:870-878 2017 (Bind m^{6}A probe in HeLa cytoplasm)
RPA1	Edupuganti et.al Nat Struct Mol Biol 24:870-878 2017 (Bind m^{6}A probe in mNPC cytoplasm)
RPA2	Edupuganti et.al Nat Struct Mol Biol 24:870-878 2017 (Bind m^{6}A probe in mNPC cytoplasm)
RPA3	Edupuganti et.al Nat Struct Mol Biol 24:870-878 2017 (Bind m^{6}A probe in mNPC cytoplasm)
SF3B4	Arguello et.al *J Am Chem Soc* 139: 17249-17252 2017 (Bind m6A probe in HeLa lysate)<br>Edupuganti et.al Nat Struct Mol Biol 24:870-878 2017 (Bind m^{6}A probe in HeLa cytoplasm)
SSBP1	Koh et.al Nucleic Acids Res. 46:11659-11670 2018 (Bind m^{6}dA probe in HEK293T lysate)<br>Edupuganti et.al Nat Struct Mol Biol 24:870-878 2017 (Bind m^{6}A probe in HeLa cytoplasm)
TSNAX	Edupuganti et.al Nat Struct Mol Biol 24:870-878 2017 (Bind m^{6}A probe in HeLa cytoplasm)
ZCCHC8	Edupuganti et.al Nat Struct Mol Biol 24:870-878 2017 (Bind m^{6}A probe in HeLa cytoplasm)
